# Supplementary material for: A framework to efficiently describe and share reproducible DNA materials and construction protocols
Source: Nat Commun. 2022 May 24;13:2894. doi: 10.1038/s41467-022-30588-x (PMC9130275; doi:10.1038/s41467-022-30588-x)
Supplement: Supplementary file 1 — Supplementary Information [file 41467_2022_30588_MOESM1_ESM.pdf]

A framework to efficiently describe and share reproducible DNA materials and construction protocols

Hideto Mori[1–3] and Nozomu Yachie[1,4,5]*

[1]Research Center for Advanced Science and Technology, The University of Tokyo, Tokyo 153-8904, Japan. [2]Institute for Advanced Biosciences, Keio University, Tsuruoka 997-0035, Japan. [3]Graduate School of Media and Governance, Keio University, Fujisawa, Kanagawa 252-0882, Japan. [4]School of Biomedical Engineering, Faculty of Applied Science and Faculty of Medicine, The University of British Columbia, Vancouver, BC V6T 1Z3, Canada. [5]Twitter: @yachielab

*Correspondence should be addressed to N.Y. (nozomu.yachie@ubc.ca).

**Supplementary Figure 1**	Operational process chart for pCMV-Target-ACEmax
**Supplementary Figure 2**	Operational process charts of QUEEN-generated plasmids derived by recycling previously established DNA objects
**Supplementary Figure 3**	Operational process charts of QUEEN-generated plasmids derived by recycling previously generated protocols
**Supplementary Figure 4**	Operational process histories of the BLADE’s 6-input, 1-output Boolean logic LUT
**Supplementary Table 1**	List of QUEEN scripts demonstrated in this study
**Supplementary Table 2**	List of base editor expression plasmid maps generated using QUEEN
**Supplementary Table 3**	Output DNA maps for the BLADE's 6-input, 1-output Boolean logic LUT

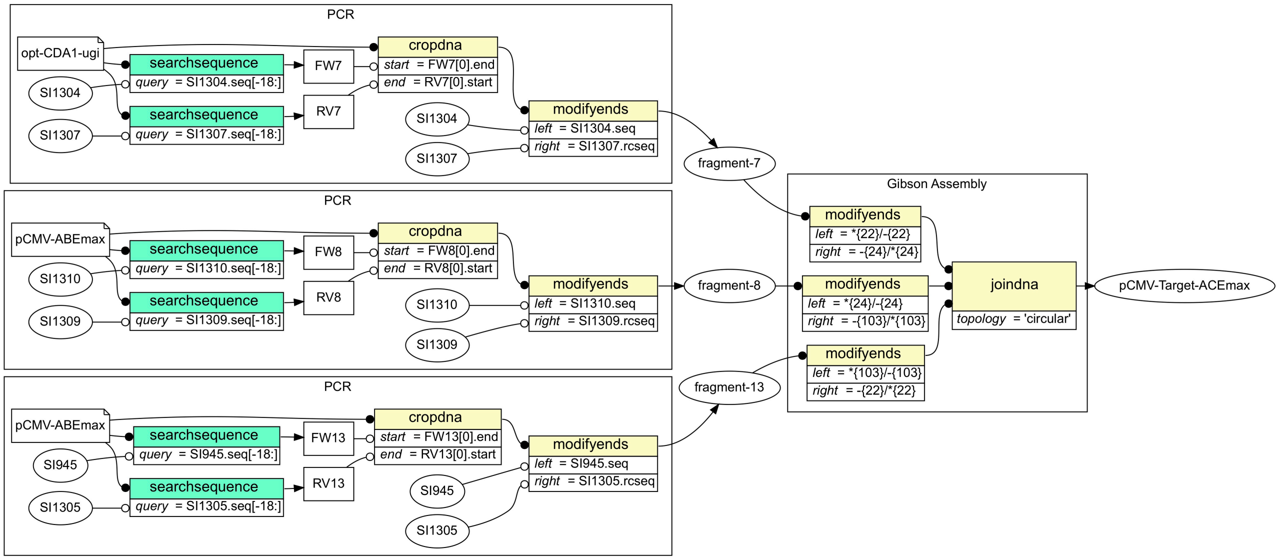

Supplementary Figure 1

Operational process chart for pCMV-Target-ACEmax. The visualization was directly generated from the output gbk file using QUEEN. The file shape, round and uncolored rectangular objects represent the input gbk files, dsDNA objects and feature objects, respectively. Colored boxes represent QUEEN operational functions with the colors corresponding to Fig. 2. Open and closed circle-headed lines represent information flows as QUEEN objects and input parameters, respectively.

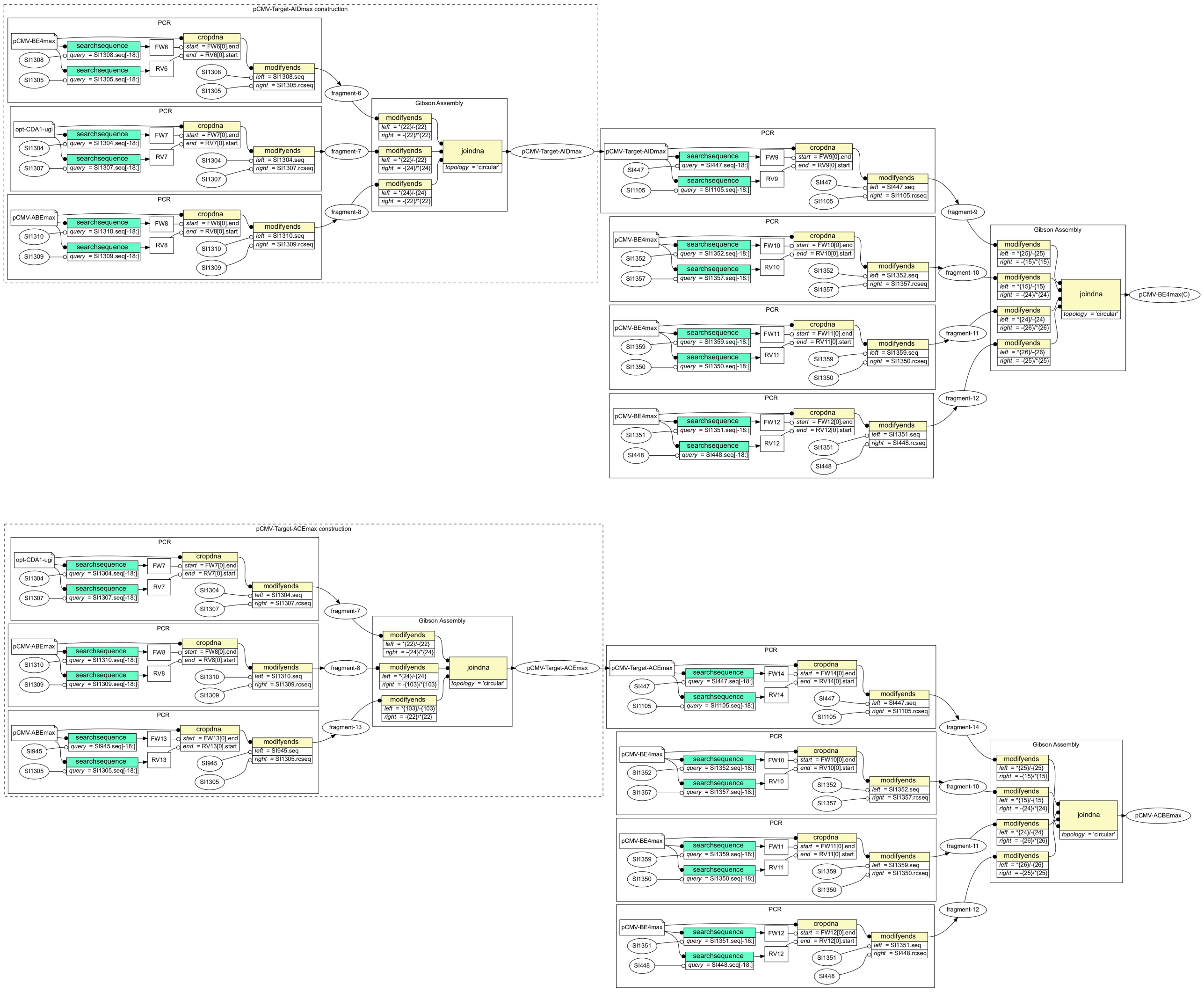

Supplementary Figure 2

Operational process charts of QUEEN-generated plasmids derived by recycling previously established DNA objects. The visualizations were directly generated from the respective product gbk files using QUEEN. The file shape, round and uncolored rectangular objects represent the input gbk files, dsDNA objects and feature objects, respectively. Colored boxes represent QUEEN operational functions with the colors corresponding to Fig. 2. Open and closed circle-headed lines represent information flows as QUEEN objects and input parameters, respectively. The operational histories enclosed by the dashed lines represent those of DNA objects previously generated by QUEEN and imported for the present DNA constructions.

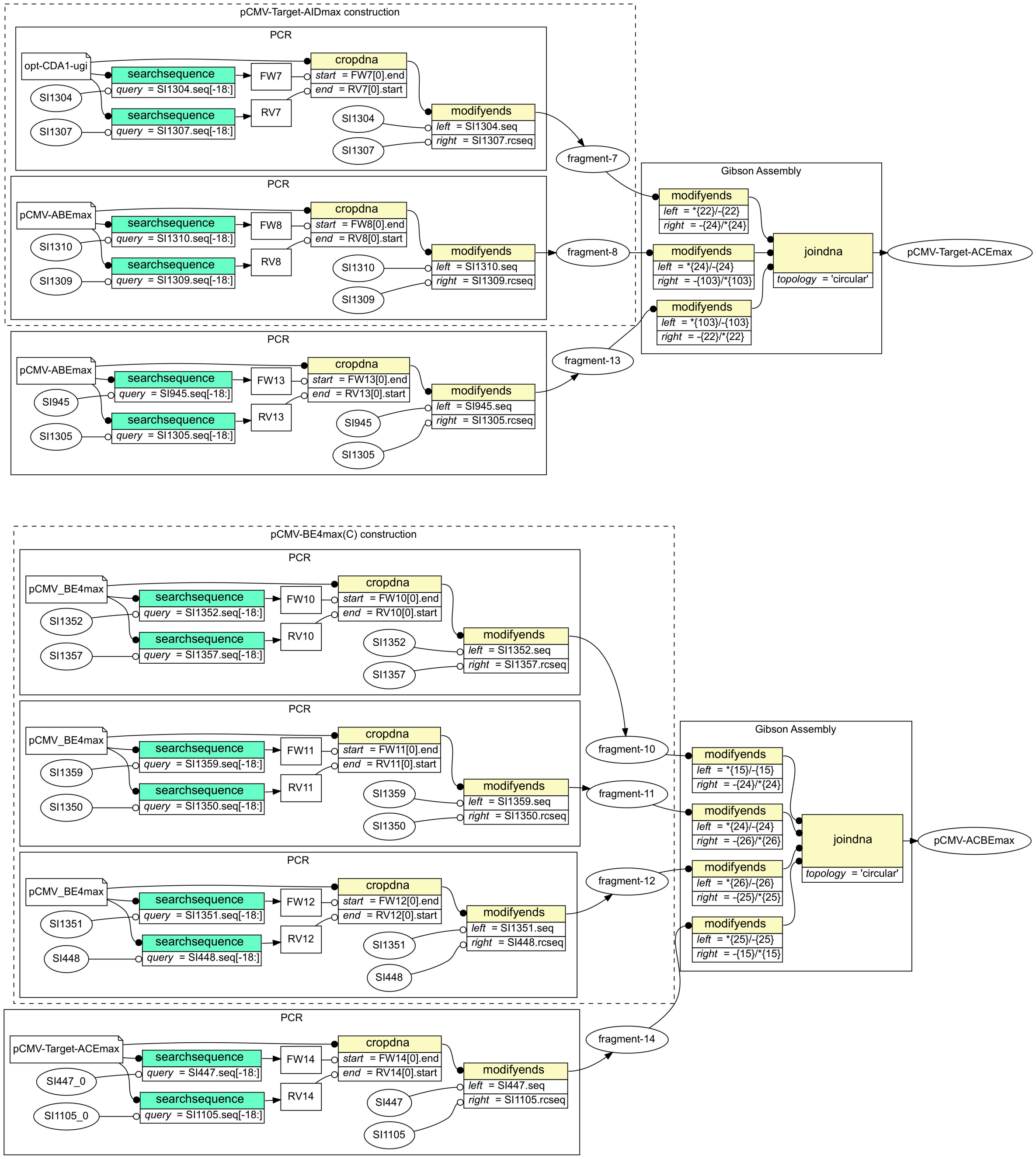

Supplementary Figure 3

Operational process charts of QUEEN-generated plasmids derived by recycling previously generated protocols. pCMV-Target-ACEmax and pCMV-ACBEmax were demonstrated to also be generated by recycling of protocols obtained from gbk files previously established using QUEEN. The visualizations were directly generated from the respective product gbk files using QUEEN. The file shape, round and uncolored rectangular objects represent the input gbk files, dsDNA objects and feature objects, respectively. Colored boxes represent QUEEN operational functions with the colors corresponding to Fig. 2. Open and closed circle-headed lines represent information flows as QUEEN objects and input parameters, respectively. The operational histories enclosed by the dashed lines represent those recycled from the previously generated QUEEN objects.

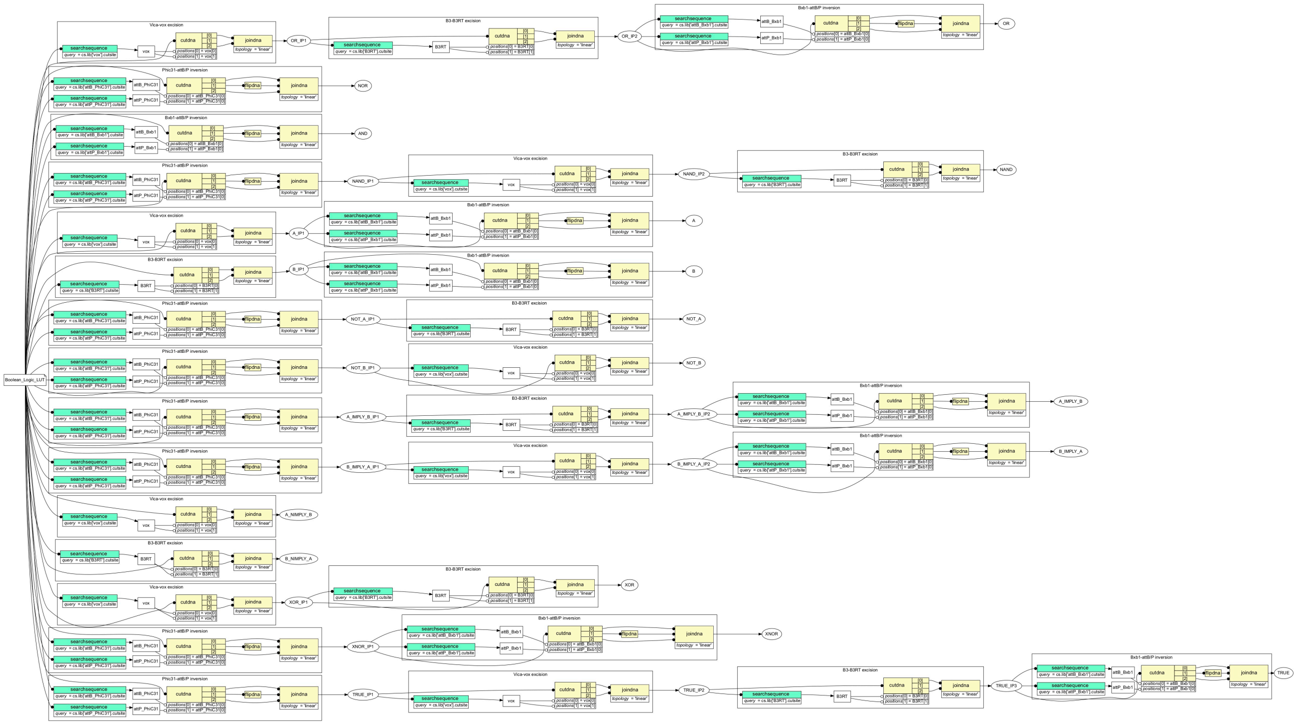

Supplementary Figure 4

Operational process histories of the BLADE's 6-input, 1-output Boolean logic LUT. The input LUT circuit was simulated for each of the 64 input signal patterns. The visualization was directly generated from the respective product gbk files using QUEEN. The file shape, round and uncolored rectangular objects represent the input gbk files, dsDNA objects and feature objects, respectively. Colored boxes represent QUEEN operational functions with the colors corresponding to Fig. 2. Open and closed circle-headed lines represent information flows as QUEEN objects and input parameters, respectively.

Supplementary Table 1. List of QUEEN scripts demonstrated in this study.

File name	Description	Jupyter Notebook	Google Colaboratory
tutorial_ex01-23.ipynb	Example QUEEN scripts 1-23	https://github.com/yachielab/QUEEN/blob/master/demo/tutorial/tutorial_ex01-23.ipynb	https://colab.research.google.com/drive/1ubN0O8SKXUr2t0pecu3I6Co8ctjTp0PS?usp=sharing
tutorial_ex24-28.ipynb	Example QUEEN scripts 24-28	https://github.com/yachielab/QUEEN/blob/master/demo/tutorial/tutorial_ex24-28.ipynb	https://colab.research.google.com/drive/1dPcNhsOl2sne_wq7ZULXXFUxizR6JQrR?usp=sharing
pRS112_construction.ipynb	QUEEN script for pRS112	https://github.com/yachielab/QUEEN/blob/master/demo/sakata_et_al_2020/pRS112_construction.ipynb	https://colab.research.google.com/drive/1x3qk7gpsuAuT_ryCPxvu6CtaI7y_slrM?usp=sharing
pCMV_Target_AID_construction.ipynb	QUEEN script for pCMV-Target-AID	https://github.com/yachielab/QUEEN/blob/master/demo/sakata_et_al_2020/pCMV_Target_AID_construction.ipynb	https://colab.research.google.com/drive/1qtgYTJuur0DNr6atjzSRR5nnjMsJXv_9?usp=sharing
pCMV_Target_ACE_construction.ipynb	QUEEN script for pCMV-Target-ACE	https://github.com/yachielab/QUEEN/blob/master/demo/sakata_et_al_2020/pCMV_Target_ACE_construction.ipynb	https://colab.research.google.com/drive/1JimBfiMIiGn1jaYK91MtRDzzGOMjz2vf?usp=sharing
pCMV_Target_AIDmax_construction.ipynb	QUEEN script for pCMV-Target-AIDmax	https://github.com/yachielab/QUEEN/blob/master/demo/sakata_et_al_2020/pCMV_Target_AIDmax_construction.ipynb	https://colab.research.google.com/drive/1f3hiUK422a9pS0qFSkuJd4jcJvOTktqW?usp=sharing
pCMV_Target_ACEmax_construction.ipynb	QUEEN script for pCMV-Target-ACEmax	https://github.com/yachielab/QUEEN/blob/master/demo/sakata_et_al_2020/pCMV_Target_ACEmax_construction.ipynb	https://colab.research.google.com/drive/1HwYTTnjQ-mufRpdb7Tn7NT9IpCj_-G9L?usp=sharing
pCMV_BE4maxC_construction.ipynb	QUEEN script for pCMV-BE4max(C)	https://github.com/yachielab/QUEEN/blob/master/demo/sakata_et_al_2020/pCMV_BE4maxC_construction.ipynb	https://colab.research.google.com/drive/1VIUltj8VHu2J6D65Iye5djrJp3kuFxv5?usp=sharing
pCMV_ACBEmax_construction.ipynb	QUEEN script for pCMV-ACBEmax	https://github.com/yachielab/QUEEN/blob/master/demo/sakata_et_al_2020/pCMV_ACBEmax_construction.ipynb	https://colab.research.google.com/drive/1ZR-Yq5lC9bkAf-Y4EV808jYCUJDvajvm?usp=sharing
pCMV_Target_ACEmax_construction_v2.ipynb	QUEEN script for the construction of pCMV-Target-ACEmax by editing the quine code obtained from pCMV-Target-AIDmax.gbk	https://github.com/yachielab/QUEEN/blob/master/demo/sakata_et_al_2020/pCMV_Target_ACEmax_construction_v2.ipynb	https://colab.research.google.com/drive/1MPcL4P71PpWN9_kbQoofFZtLER0WXL9Y?usp=sharing
pCMV_ACBEmax_construction_v2.ipynb	QUEEN script for the construction of pCMV-ACBEmax by editing the quine code obtained from pCMV-BE4max(C).gbk	https://github.com/yachielab/QUEEN/blob/master/demo/sakata_et_al_2020/pCMV_ACBEmax_construction_v2.ipynb	https://colab.research.google.com/drive/1atdnNEJQPS_BiZ66SuF3nrYTv2xfXxBw?usp=sharing
Boolean_logic_LUT.ipynb	QUEEN script for simulating sequence state alternations in the BLADE Boolean logic LUT	https://github.com/yachielab/QUEEN/blob/master/demo/Weinberg_et_aL_2017/Boolean_logic_LUT.ipynb	https://colab.research.google.com/drive/1sQNhWYVNXzROLQAfpzoJpzQkNLHxidT8?usp=sharing

Supplementary Table 2. List of base editor expression plasmid maps generated using QUEEN.

Plasmid name	Benchling
pCMV-Target-AID	https://benchling.com/s/seq-zBfAxztMQhd0QTsbwLXo?m=slm-yfHLxjSWcPNfZygZlwAe
pCMV-Target-ACE	https://benchling.com/s/seq-xXRIehES9wMRdBOVoxl3?m=slm-44NLCXaxvGjp62NNx8ti
pCMV-Target-AIDmax	https://benchling.com/s/seq-z5HofLURIU2LtWtmqjYS?m=slm-dkF9dANDILZzTE8jdio7
pCMV-Target-ACEmax	https://benchling.com/s/seq-70uRXXVhQiANrwkhIReK?m=slm-9cOQEy1QQppXpyDIxyzI
pCMV-BE4maxC	https://benchling.com/s/seq-fbeQv0gpuSYuf43xEDMs?m=slm-pKeRdE4FJeS8nzXFKSoL
pCMV-ACBEmax	https://benchling.com/s/seq-18yINK7lOT20h7yWYBb8?m=slm-Ky8vntrJ82hf0baubppp

Supplementary Table 3. Output DNA maps for the BLADE's 6-input, 1-output Boolean logic LUT.

Input		Circuit configurators					Ouput
A (Cre)	B (Flp)	S1 (PhiC31)	S2 (Vilka)	S3 (B3)	S4 (Bxb1)	Logic gate	Sequence (Benchling URL)
0	0	0	1	1	1	OR	https://benchling.com/s/seq-yz8xUZtqXvQrH1HjQD1I?m=slm-2v9hN8paG7ZbiH1vYcEi
1	0						https://benchling.com/s/seq-fvAQW0zwITAJQewdlUrP?m=slm-ypaRQxRSpETsi5oqE228
0	1						https://benchling.com/s/seq-KnNwG6trQvRKbCpcWLow?m=slm-95QOgoBQcWvxnG6wHSc3
1	1						https://benchling.com/s/seq-KzedTWo615vO9sPFTq45?m=slm-HvlSL8zfY8bUpYrhGxbW
0	0	1	0	0	0	NOR	https://benchling.com/s/seq-mggCd1J8ZiO9MDOMfrGa?m=slm-O7ZENhhmR6a6chVyz4k2
1	0						https://benchling.com/s/seq-p3Z17SHc2ZzIpXCXdhJ8?m=slm-yAxO3VMkr1cXF1RTPWEZ
0	1						https://benchling.com/s/seq-PI9pq7D1XRNxkf0AP2Do?m=slm-Z0yE9E1BgrGkk0jfLsuF
1	1						https://benchling.com/s/seq-NiK7PTixd1PR2pF61W8F?m=slm-G1dhbiqrLPMEgc6iVFEC
0	0	0	0	0	1	AND	https://benchling.com/s/seq-5kdr3IDda7TjwLuVjG30?m=slm-mzVW5FRJlR3P4sutcDOa
1	0						https://benchling.com/s/seq-1fQbFXJHkvbeaLLzXMb4?m=slm-8TQvwcRPSiLswLpgpgDI
0	1						https://benchling.com/s/seq-PNPLZwSe0x9xXPLPTwM4?m=slm-D4V8SaqhCeN5DEM0sJtA
1	1						https://benchling.com/s/seq-dxz9o7PjQQUUoGGd7yJM?m=slm-zgFxy22swhjN3i0tl9yv
0	0	1	1	1	0	NAND	https://benchling.com/s/seq-f9joITYoptz6dlQczdc2?m=slm-BaHdG1fl7VQ0asgdjJDD
1	0						https://benchling.com/s/seq-Bh9j2wagu8FHOA0l3W5A?m=slm-vhnJ3feenyzDCjT7ONy1
0	1						https://benchling.com/s/seq-UVhUxs70ijRLSXdnpxD8?m=slm-KtG15S8dyBPBkS4JUtzi
1	1						https://benchling.com/s/seq-4IgU1s3KVcp9OeQRyvfd?m=slm-uYDjDrVjXulKmeSCXLfF
0	0	0	1	0	1	A	https://benchling.com/s/seq-KrKLtfa2UvCKJbhWuz7e?m=slm-Phza8BYFOQ2fdu9J1U6f
1	0						https://benchling.com/s/seq-abanZpUNkMOF4eNcx1CL?m=slm-2MXqugDm6aIaRquSYWGK
0	1						https://benchling.com/s/seq-HQ7JbkMJL6jGGzJxDdn0?m=slm-81dVD0RQ6ZyLBg7m9Nwm
1	1						https://benchling.com/s/seq-pcTFEuNOZlQjYli2edPa?m=slm-iZAtPmLrtAJiV7qFf5E6
0	0	0	0	1	1	B	https://benchling.com/s/seq-vKRkkDak38ptE0coM7FU?m=slm-DQx2RSxFptGSyinkg0v0
1	0						https://benchling.com/s/seq-AB1zbtkCLLpEuugSaQih?m=slm-WMA8hpE7IortVcZFoX5i
0	1						https://benchling.com/s/seq-dIXc0LYcHnQVctf4jxli?m=slm-7MGVQ7dvugGTvv9htiap
1	1						https://benchling.com/s/seq-xvhmpq1Axft2jN1YaKfY?m=slm-0nsh6kpyM5fcneoUVfp7
0	0	1	0	1	0	NOT A	https://benchling.com/s/seq-rdu9QNvk2AFPKuomSgKI?m=slm-vf45nKAdglkZxQs64VtP
1	0						https://benchling.com/s/seq-cYdRjVRUpzT4GUIJJ3Dd?m=slm-qRC4TsE2WhrQ5Ud8kLng
0	1						https://benchling.com/s/seq-85lrXatLB00Pp83yfwjz?m=slm-eEwJbbbOVQ9ddMlof74o
1	1						https://benchling.com/s/seq-pmMO2aV2gXPWMooyJiNQ?m=slm-QZN6KJTCbjreDr2OwKqg
0	0	1	1	0	0	NOT B	https://benchling.com/s/seq-0xjzKvycR48EP62fhvmD?m=slm-trChWieZTy59DyooD3kd
1	0						https://benchling.com/s/seq-XtpLcQ72d23dh1zZX1S7?m=slm-9Gyar16ByaqRPcdbQ4eJ
0	1						https://benchling.com/s/seq-XtpLcQ72d23dh1zZX1S7?m=slm-9Gyar16ByaqRPcdbQ4eJ
1	1						https://benchling.com/s/seq-r6yN12tzq0fRTieNxoPu?m=slm-A5PTiqnMDEFbEV3UXWUJ
0	0	1	0	1	1	A IMPLY B	https://benchling.com/s/seq-sqk0m4HQp3oHOep0EgzY?m=slm-18IWwjp4hed65uVVxD7y
1	0						https://benchling.com/s/seq-3aSP2oL5wPPue1NDVmli?m=slm-VLgHW78jmzDNJ5Zlkac3
0	1						https://benchling.com/s/seq-ouvbeg99Lzacgvl8gzWc?m=slm-wmaZhCNf4IKmoqp3a5qy
1	1						https://benchling.com/s/seq-ueuvXAeFzYCC5YtDRWRW?m=slm-6aHfMKbtTShaiynAvaho
0	0	1	1	0	1	B IMPLY A	https://benchling.com/s/seq-yTUoHCy4eAJFcRETtrF0?m=slm-Z7AfoP0L86ERTZ1mLkbh
1	0						https://benchling.com/s/seq-63hyauQ78JboKRkYHrvL?m=slm-wTlpw1uFhNtPKonuqpGp
0	1						https://benchling.com/s/seq-DDKqI5thmPDg7g9BYSTA?m=slm-PHK208sZvq2yyP0JVLKy
1	1						https://benchling.com/s/seq-h3GzylbQXX1mOIZ6Sri5?m=slm-nM0RoR6txKH3DCqHMjUc
0	0	0	1	0	0	A NIMPLY B	https://benchling.com/s/seq-Hh8mnhbuHTX0bneLorUX?m=slm-cJR6dNpI5o3v89Dk3oPT
1	0						https://benchling.com/s/seq-5k60ZEsBux2QpnUKgjhw?m=slm-YQOJCLY1abza84RQ6KZA
0	1						https://benchling.com/s/seq-4kjD165lNIuHm16w8JoH?m=slm-RlbZsbQw0ROAPMjRsCls
1	1						https://benchling.com/s/seq-4ajVA7HvkPsCung7uRub?m=slm-CS2qZwNAgALKa9gNMbd9
0	0	0	0	1	0	B NIMPLY A	https://benchling.com/s/seq-2nLVpCo10m9rTjLyVDqT?m=slm-RT5O0LCRYM87zE0CAp3a
1	0						https://benchling.com/s/seq-31pmQb2OJufJHvpMKjEM?m=slm-zFm4Lat1tF0qytWmofZD
0	1						https://benchling.com/s/seq-5k60ZEsBux2QpnUKgjhw?m=slm-YQOJCLY1abza84RQ6KZA
1	1						https://benchling.com/s/seq-3Ot5N3PJOv4hC4FIVifP?m=slm-cjWOCaz0QnaH4sRF9NMj
0	0	0	1	1	0	XOR	https://benchling.com/s/seq-bs0ft5Cwc3zWGlCh6gCJ?m=slm-f8ABZkX4PXAZzvmla3Ei
1	0						https://benchling.com/s/seq-bdIdHusM1BvRclxMvx8b?m=slm-KoGsqPK2mnPA3QiKUWgJ
0	1						https://benchling.com/s/seq-rocX0Rq7QP3cCm8iDyYv?m=slm-TGDbXyL8JlfKSVvXtmfb
1	1						https://benchling.com/s/seq-X4R4Q3ABwdQaWYcBb261?m=slm-DL8IqgSS4dZ1bexFCPnx
0	0	1	0	0	1	XNOR	https://benchling.com/s/seq-kQlKifOrR9kPHkD9KjyW?m=slm-u6dbCxBgDZrXwpYNqAKf
1	0						https://benchling.com/s/seq-77l1m4KTsnlj89xYwlIo?m=slm-Td5BgG7wHcMlIp57oUPP
0	1						https://benchling.com/s/seq-oFHU2jhMwcvMpykJWkhG?m=slm-mUmcfmnl8H3cep4WLeME
1	1						https://benchling.com/s/seq-ffVc3KorfByzBKJyYoVe?m=slm-XO4GOsX4p5M405i3aIE3
0	0	1	1	1	1	TRUE	https://benchling.com/s/seq-FzdMRynpcKIn615na2Pd?m=slm-lBXyIXv1wI7XvdzI1IBe
1	0						https://benchling.com/s/seq-KJIpoFRHRu3QGUfywGrl?m=slm-2WwOxT37MjYRY83wbmaY
0	1						https://benchling.com/s/seq-JYt5CkeDbF2yJOe6wK2Z?m=slm-qEcBYqi6oGSiModDCknf
1	1						https://benchling.com/s/seq-0aHARFroFilMq0fqXwgr?m=slm-7oiufVZJU4Pk0gssbnYl
0	0	0	0	0	0	FALSE	https://benchling.com/s/seq-S3m92nDznr3cPEfyrClp?m=slm-VhBBYVnO66y9muPkM5cb
1	0						https://benchling.com/s/seq-tSWjXbZoP5sZBzPmPDVw?m=slm-ITSDQsMkhrPThIs9BkEN
0	1						https://benchling.com/s/seq-JPFsCvagXmer6mWzLe4h?m=slm-zQYhPdgl46eUvu0A8dOe
1	1						https://benchling.com/s/seq-v5FQugy5EoVQ251AvNwk?m=slm-zDABXVCzSOzkL2q5EYhE
